# Ketogenic Diet as a Possible Non-pharmacological Therapy in Main Endocrine Diseases of the Female Reproductive System: A Practical Guide for Nutritionists

**DOI:** 10.1007/s13679-023-00516-1

**Published:** 2023-07-05

**Authors:** Elisabetta Camajani, Alessandra Feraco, Ludovica Verde, Eleonora Moriconi, Marco Marchetti, Annamaria Colao, Massimiliano Caprio, Giovanna Muscogiuri, Luigi Barrea

**Affiliations:** 1 Department of Human Sciences and Promotion of the Quality of Life, San Raffaele Roma Open University, 00166 Rome, Italy; 2 Laboratory of Cardiovascular Endocrinology, IRCCS San Raffaele, Rome, Italy; 3 Centro Italiano Per La Cura E Il Benessere del Paziente Con Obesità (C.I.B.O), Dipartimento Di Medicina Clinica E Chirurgia, Unit of Endocrinology, Università Degli Studi Di Napoli Federico II, Naples, Italy; 4 Department of Public Health, University “Federico II” of Naples, 80138 Naples, Italy; 5 Section of Clinical Nutrition and Nutrigenomic, Department of Biomedicine and Prevention, University of Tor Vergata, Via Montpellier 1, 00133 Rome, Italy; 6 Dipartimento Di Medicina Clinica E Chirurgia, Unità Di Diabetologia E Andrologia, Università Degli Studi Di Napoli Federico II, Via Sergio Pansini 5, 80131Naples, Endocrinologia, Italy; 7 Cattedra Unesco “Educazione Alla Salute E Allo Sviluppo Sostenibile”, Università Degli Studi Di Napoli Federico II, Naples, Italy; 8 Dipartimento Di Scienze Umanistiche, Centro Direzionale, Università Telematica Pegaso, Via Porzio Isola F2, 80143 Naples, Italy

**Keywords:** Ketogenic diets, VLCKD, PCOS, Endocrine diseases, Female reproductive, Nutritionists

## Abstract

**Purposeof Review:**

This narrative review explored the role of ketogenic diets (KDs) in improving fertility outcomes, low-grade inflammation, body weight, visceral adipose tissue, and its potential use in certain types of cancer, through its favorable actions on mitochondrial function, reactive oxygen species generation, chronic inflammation, and tumor growth inhibition.

**Recent Findings:**

Nutrition is crucial to maintain the female reproductive system’s health. Evidence on the association between diet and female reproductive system has greatly expanded over the last decade, leading to the identification of specific diet therapy, particularly KDs. KDs has been proved to be an effective weight-loss tool. To date, KDs is being increasingly used in the treatment of many diseases, such as obesity, type 2 diabetes mellitus. KDs is a dietary intervention capable of ameliorating the inflammatory state and oxidative stress through several mechanisms.

**Summary:**

Due to the increasing use of KDs beyond obesity, this literature review will provide the latest scientific evidence of its possible use in common disorders of the female endocrine-reproductive tract, and a practical guide to its use in these patients.

## Introduction

Obesity is responsible for several disorders affecting the female reproductive tract, many of which lead to infertility, such as polycystic ovary syndrome (PCOS) and endometriosis [1–3]. In particular, obesity displays a negative effect on women’s reproductive health, predisposing the development or exacerbation of endocrine disorders of the reproductive tract [2, 4]. Visceral adipose tissue expansion, hyperinsulinemia, and low-grade chronic inflammation resulting from obesity, contribute to the harmful and detrimental dysregulation of reproduction and the subsequent development of fertility disturbances [2]. In this regard, both PCOS and endometriosis share a chronic inflammatory status, which is fueled in the presence of obesity [1, 3, 5]. In addition, chronic inflammation cooperates with insulin resistance and hyperandrogenism to constitute an interactive continuum acting on the pathophysiology of PCOS [3].

Moreover, certain female reproductive tract disorders are more common in women with obesity, such as breast and endometrial cancer: one of the links between obesity and female cancers involves, for instance, impaired glucose metabolism, insulin resistance, and hyperinsulinism [6–8]. Obesity has been showed to increase the risk of developing breast cancer, especially for the molecular subtype triple negative breast cancer, and to worsen the prognosis [9]. Of note, breast cancer diagnosed before the menopause shows a more aggressive phenotype, and there is consistent interest in promoting prevention dietary strategies in order to reduce the incidence of this neoplasia in premenopause [10].

Finally, menopause, the physiological cessation of reproductive capacity in a woman’s life, tends to be associated with an increased risk of obesity and a shift toward a distribution of abdominal fat with a relative increase in health risks [11, 12]. Changes in body composition during menopause may be caused by a decrease in circulating estrogens, and the relative increase in androgen-to-estrogens ratio, related to altered fat distribution [11, 12]. Weight gain and increased visceral fat distribution associated with menopausal transition, are responsible for secretion of pro-inflammatory adipocytokines, which leads to type 2 diabetes (T2DM) and cardiovascular diseases (CVD) [13, 14]. In this context, dietary strategies to prevent obesity and obesity-related diseases in menopausal women, including sleep disturbances that represent one of the main symptoms of menopause, should be one of the main objectives for Nutritionists [13, 15].

It is clear that in main endocrine disorders of the female reproductive tract, treatment of excess weight is imperative. Optimal management of obesity requires a multidisciplinary approach to promote weight loss, which can reduce its detrimental health effects [16]. Currently, several approaches to weight loss are available, including different dietary treatments, cognitive behavioral interventions, pharmacological therapies, and surgical interventions [17, 18]. Despite this range of weight loss strategies, less than 20% of individuals who try to lose weight are able to achieve and maintain a 10% reduction over a year, with the majority gaining it back within 3–5 years [19]. As individuals regain weight, many of the associated comorbidities have the potential to return, leading to a great deal of frustration and feelings of hopelessness.

According to Trimboli, in recent years there has been confusion in relation to the nomenclature of ketogenic diets (KDs) [20]. The authors clarify that very low-calorie ketogenic diet (VLCKD) is a KD with a very low-calorie content (< 800 kcal), with 30–50 g of carbohydrates, normoproteic (1.2–1.5 g pro kg), hypolipidic (about 20–40 g/die). On the other hand, low-calorie ketogenic diet (LCKD) is a KD with a calorie content > 800 kcal and < total energy expenditure (TEE), with 30–50 g of carbohydrates, normoproteic and with a lipid content > 40 g. Lastly, the KD or iso-calorie ketogenic diet (ICKD) includes a calorie content equal to TEE, 30–50 g of carbohydrates and about 70–80% of lipids [21–23]. The KD has been used primarily for the treatment of therapy-resistant epilepsy in children [24]. Of note, more restrictive ketogenic approaches, such as the VLCKD, have attracted attention not only for rapid weight loss but also for improved endocrine features, cardiometabolic risk profile, and reproductive function [25–27]. From an endocrine and reproductive perspective, its success in patients with endocrine diseases of the female reproductive tract and obesity may be due to some fundamental aspects: rapid and effective weight loss, particularly of fat mass (FM) and visceral adipose tissue, its antioxidant and anti-inflammatory properties and the reduction of insulin resistance [28–31].

Given the increasing use of VLCKD not only in obesity but also in other related diseases, this literature review will provide the latest scientific evidence of its possible use in the female endocrine-reproductive field as well, and a practical guide to its use in these patients. In this narrative review, we will firstly define and classify obesity, noting which methods are most widely used for body composition analysis. Subsequently, we will explore the beneficial effects of KDs in women life stages, such as the fertile and reproductive period, as well as menopause. Afterwards, we will investigate the potential benefits of the KDs in major disorders of the female reproductive system, such as PCOS, and the potential use of the KDs in the most common cancers of the female reproductive system, such as breast and endometrial cancer.

## Definition of Obesity, its Classification and Methodologies to Detect Body Composition

The World Health Organization (WHO) defines obesity as a chronic condition characterized by excess body fat that can cause medical, psychological, physical, social and economic problems and in 1997 declared obesity as a major public health problem and a global epidemic [32]. The prevalence of adult overweight and obesity has increased worldwide since the 1980s, with no decrease in any country during the 33 years of recorded data [33]. Of note, obesity prevalence is increasing among adolescents and children, thus representing a serious public health problem, affecting all demographic groups to a greater or lesser extent. Considering the alarming increase of subjects with overweight and obesity, the WHO has coined the term ‘*Globesity*’ to describe this phenomenon that has reached the numbers of a global epidemic. Among adults, the prevalence of obesity was generally higher for women than for men in all age brackets. Obesity has become a major contributor to the global burden of chronic diseases affecting virtually all age and socio-economic groups worldwide [34, 35]. Furthermore, it is now recognized that obesity increases the risk of multiple metabolic diseases such as hyperlipidemia, insulin resistance, T2DM, hypertension, atherosclerosis and cardiovascular complications; however, obesity is also correlated with rising infertility and an increase of endocrine disorders including endometriosis and PCOS [36].

## Body Composition Assessment

Body mass index (BMI) is the assessment tool used worldwide to estimate the degree of overweight or obesity [37]. According to BMI, individuals are allocated to five different categories as: 18.5–24.9 kg/m^2^ for normal range, 25.0–29.9 kg/m^2^ for overweight, 30.0–34.9 kg/m^2^ for class 1-obesity, 35.0–39.9 kg/m^2^ for class 2-obesity and equal or greater than 40.0 kg/m^2^ for class 3-obesity [38]. However, measuring BMI alone has proven inadequate to help clinicians to assess and manage obesity-related health risks in their patients; in fact, BMI does not provide indications about individual’s body composition and adiposity distribution, and today it is well established that abdominal obesity is correlated with increased cardiovascular risk, T2DM, and even some female cancers [37, 39–41].

Adipose tissue distribution differs between genders. Men mainly tends to accumulate visceral fat, resulting in the classical android body shape, which is highly correlated with increased cardiovascular risk. On the other hand, women display higher percent body fat and deposit it in a different pattern, with more adipose tissue in the subcutaneous depot, before menopause, a characteristic that offers protection from the negative consequences associated with obesity and metabolic syndrome [42]. After menopause, fat deposition and accumulation shift in favor of visceral deposition. Such modification is accompanied by a parallel increase in metabolic risk reminiscent of that observed in men [42]. Therefore, evaluation of body composition is primarily important to assess the health risk of individuals with obesity and subsequently to target and tailor nutritional treatment.

Several methods can be used to evaluate body composition, with different outcomes on precision and accuracy.

Anthropometric measurements are noninvasive quantitative measurements including height, weight, body circumferences and skinfold thickness [43].

Waist circumference (WC) is a simple method of assessing abdominal obesity that is easily standardized and clinically applicable. According to the National Center for Health Statistics (NCHS), WC should be measured at the tightest point or, when the tightest point is not visible, at an intermediate level between the lower edge of the rib cage and the iliac using a non-stretchable measuring tape [44]. Patients should stand with feet together, on a plane parallel to the ground, with their hands on hips. During the assessment, the patient should breathe normally, and the tape might not compress the skin and should be parallel to the floor [44]. Current guidelines recommend using a single WC threshold for Caucasian women > 88 cm to indicate high WC and increased cardiovascular and T2DM risk [45, 46]. In addition, from the combination of WC and hip circumference, the waist to hip ratio (WHR) can be obtained, the most commonly used index of abdominal obesity. For women, the WHO states that abdominal obesity is defined as a WHR above 0.85 [47]. Thus, WC allows further refinement of the adverse health risk characterized by BMI, and this measurement should be included in stratification of health risk associated with obesity. Resistance to the routine inclusion of WC in clinical practice not only ignores evidence of its utility but also fails to take advantage of the opportunity to counsel patients regarding the higher-risk phenotype of obesity. In addition, measurement of both BMI and WC provides unique opportunities to track the benefits of treatment and the efficacy of interventions for obesity and related metabolic diseases.

Skinfold thicknesses measure subcutaneous body fat at various regions of the body, including triceps, biceps, subscapularis, and suprailiac [48]. Equations are available to calculate total body fat from these measurements (usually for research purposes). The exact technique may vary, but in general the measurement of skinfolds is achieved by grasping the skin 2 cm away from the measurement site [48]. Despite standard measurement techniques, skinfold testing has high variability and has had limited use in clinical settings to date. In addition, skinfold measurement in individuals with obesity is difficult and rarely used [49]. In fact, particularly in subjects with severe obesity, the skinfold may be larger than the caliper can measure; the fold of skin and fat compresses with repeated measurements; and careless use of the caliper causes pain, bruising, and damage to the subjects’ skin [49].

Imaging techniques (dual-energy x-ray absorptiometry, computed tomography, magnetic resonance, and ultrasound) are the most advanced methods for determining body composition [50]. However, they are not feasible in clinical practice because they are expensive, time-consuming, or expose patients to radiation [50].

Bioelectrical impedance (BIA) is widely used in clinical practice and research studies to determine body composition, providing information about FM, fat free mass (FFM), and body water (total, intracellular, and extracellular water), on the basis of equations used to estimate body hydration [51]. In the context of VLCKD protocol, BIA could be used to estimate body composition modifications in terms of FFM maintenance during weight loss. Importantly, monitoring muscle mass could allow early detection of muscle loss and then correction of inadequate protein intake. In addition, phase angle (PhA) has been reported to be an important parameter to monitor during VLCKD. Indeed, PhA is a BIA-derived index that can be used to identify inflammation in various clinical settings [29, 52]. Recently, in a cohort of 260 women (aged 18–69 years, BMI 25.0–50.9 kg/m^2^), a significant decrease in plasma c-reactive protein concentrations was observed after 31 days of the active phase of VLCKD, whereas the PhA increased. Interestingly, a significant inverse relationship was observed between c-reactive protein and PhA, independent of confounding factors (BMI, WC, age, and physical activity). Therefore, monitoring PhA could be a useful tool to assess changes in the inflammatory status of patients treated with VLCKD and to avoid blood draws and expensive biochemical tests [29].

## Ketogenic Diets in Women Life Stages

### Ketogenic Diets and Fertility

Infertility is defined as the failure to establish a clinical pregnancy after 12 months of regular, unprotected sexual intercourse. According to Borght and Wyns, infertility affects between 8 and 12% of couples of reproductive age worldwide [53]. The WHO estimates that 48 million couples and 186 million individuals live with infertility globally [54].

As reported by Skoracka and colleagues, female infertility contributes to 35% of overall infertility cases [55]. The main factors influencing the possibility of spontaneous conception are the age of female partner and infertility linked to endocrine dysfunction [56]. The chance of achieving a spontaneous pregnancy decreases with age before conception. The decline in female fertility begins as early as 25–30 years of age and the median age of last childbirth is 40–41 years in most study populations experiencing natural fertility [56]. Disease-related infertility can affect both sexes and be gender-specific. Factors affecting female fertility include premature ovarian failure, PCOS, endometriosis, uterine fibroids and endometrial polyps [57]. Weight before conception is a major risk factor for fertility outcomes and it is well established that weight loss improves fertility in overweight and obese women [58]. Many overweight women struggling with fertility have co-morbidities. It has been estimated that 75% of infertile women who suffer from overweight or obesity have PCOS, which in itself poses additional challenges to fertility due to disturbances in insulin resistance, sex steroid metabolism and regularity of menstrual cycles [59].

KDs appears to have positive effects as it leads to rapid weight loss, particularly of FM, thus resulting in a reduction in chronic low-grade inflammation induced by dysfunctional adipose organ. Furthermore, reduction in exogenous carbohydrate consumption induces lower insulin production, with improvement in hyperinsulinism and insulin resistance, metabolic alterations commonly observed in infertile women [60].

According to Arbour and colleagues, obesity may negatively affect different stages of woman’s reproductive life, including early menarche, fertility, pregnancy and menopause, as a result of hormonal imbalances and insulin resistance [61]. Moreover, weight loss has positive effects on fertility as well as on the chances of successful assisted reproductive technologies (ART) [62–64]. A retrospective cohort study conducted by Moragianni and colleagues showed that women with obesity are 68% less likely to have a live baby after the first cycle of ART than women without obesity [65]. Furthermore, obesity is correlated with the need for higher doses of assisted reproductive therapy drugs and lower rates of efficacy at each stage of the in vitro fertilization (IVF) process [65]. According to McGrice and Porter, weight loss can improve fertility and pregnancy outcomes, particularly with regard to IVF [58]. Benefits for this cohort of patients include more regular menstrual cycles, better quality embryos available for transfer, lower drug dosage and the need for fewer treatment cycles. In a retrospective cohort study, Russell and colleagues investigated if the nutritional components of IVF patient’s daily diet impact blastocyst development and pregnancy outcomes [66]. In this study, 120 patients completed a dietary log and had a fresh or frozen blastocyst transfer. Forty-eight patients were identified with an average daily protein intake > 25% *vs.* 72 patients who had < 25%. Patients eating > 25% proteins had significant increase in blastocyst development of 54.3% *vs.* 38.5% (*p* < 0.001) and pregnancy rates 66.6% (32/48) *vs.* 31.9% (23/72) (*p* < 0.005). Patients eating < 40% carbohydrate had a 63.2% (31/49) *vs.* 33.8% (22/71) pregnancy rate (*p* < 0.001). Protein intake of > 25% combined with < 40% carbohydrate had an 80% (29/36) pregnancy rate. According to the authors, blastocyst development with improved pregnancy rates may be directly related to nutritional components in a patient’s diet. Patients undergoing IVF with poor blastocyst development may benefit from increasing their average daily protein intake to > 25% and lowering their carbohydrate to < 40% [66].

In a prospective study of 170 women who collectively underwent 233 IVF/ICSI cycle, Chavarro and colleagues showed that overweight and obesity were related to lower live birth rates in women undergoing ART [62]. The authors confirmed that the presence of overweight or obesity at baseline was associated with lower live birth rates [adjusted live birth rate = 23% (14–36%)]. In addition, participants with obesity at baseline were associated with a lower frequency of positive β-human chorionic gonadotropin (hCG). The highest positive β-hCG, pregnancy and live birth rates were observed among women with a BMI between 20.0 and 22.4 kg/m^2^. Moreover, short-term weight loss was associated with a higher proportion of metaphase II oocytes retrieved. The adjusted proportion of metaphase II eggs was 91% (87–94%) for women who lost 3 kg or more and 86% (81–89%) for women whose weight remained stable (*p* = 0.002). This association was stronger among women with overweight or obesity at baseline [62].

When fertility represents the main target of infertile patients, particularly at more advanced ages, it is critical to rapidly improve metabolic, nutritional and endocrine conditions in order to facilitate ovulation and pregnancy.

In a systematic review by McGrice and Porter, it was confirmed that reducing carbohydrate load could reduce circulating insulin levels, improve hormonal imbalance and result in ovulation resumption to improve pregnancy rates in infertile women [58]. To this end, the authors’ findings suggest that low-carbohydrate diets may optimize fertility in some clinical groups, particularly in overweight and obese women with PCOS [58].

The application of ketogenic protocols in women with overweight or obesity and infertility is based on the possibility to reduce insulin levels, improving hormonal balance, restoring ovulatory cycles and improving oocyte and embryo quality, as well as pregnancy rates even in subjects undergoing IVF [58, 67, 68]. In particular, as already mentioned, KDs have proven higher efficacy in terms of rapidity of weight loss, dampening of insulin resistance and induction of ovulation, when compared to standard hypocaloric diets also resulting in increased sex hormone binding globulin (SHBG), reduced blood levels of free testosterone, reduced luteinizing hormone to follicle stimulating hormone (LH/FSH) ratio [69, 70].

SHBG is a glycoprotein produced by the liver that binds sex steroids with high affinity and specificity. According to Qu and Donnelly, there is a negative correlation between circulating SHBG levels and markers of insulin resistance [71]. In fact, in vitro studies indicate that SHBG may down-regulate the PI3K/AKT pathway involved in the development of local and systemic insulin resistance [72]. Decreased SHBG levels increase the bioavailability of androgens, which in turn leads to progression of ovarian pathology, anovulation and the phenotypic characteristics of PCOS. Insulin resistance is often accompanied by nutrient oversupply and high dietary intake of monosaccharides can induce low serum SHBG levels through increasing hepatic lipogenesis [73]. It has been shown that dietary fructose increases levels of the enzymes involved in de novo lipogenesis, even more strongly than high fat diet, and that fructose metabolism stimulating de novo lipogenesis is a central abnormality in NAFLD [71]. In a study conducted by Paoli and colleagues, 14 overweight women with diagnosis of PCOS underwent to KD with phytoextracts for 12 weeks [60]. The characteristic reversal of the LH/FSH ratio was observed at the beginning of the study and disappeared after 12 weeks (pre 2.00 ± 0.30 *vs* post 1.15 ± 0.20; *p* < 0.001). Compared to basal values, there was also a significant decrease in plasma concentrations of LH (pre 10.24 ± 1.43 *vs* post 6.41 ± 1.46; *p* < 0.001), total testosterone (pre 47.43 ± 6.08 ng/dL *vs* post 40.71 ± 5.77 ng/dL; *p* < 0.001), free testosterone (pre 0.96 ± 0.60 pg/mL *vs* post 0.56 ± 0.30 pg/mL; *p* = 0.009), percentage of free testosterone (pre 2.05 ± 1.33% *vs* post 2.05 ± 1.33%; *p* = 0.033) and dehydroepiandrosterone sulfate (pre 2.13 ± 0.26 μg/mL *vs* post 1.70 ± 0.20 μg/mL; *p* < 0.001). Estradiol levels were risen (pre 139.80 ± 14.93 pg/mL *vs* post 191.90 ± 38.80 pg/mL; *p* < 0.001), so did progesterone (pre 12.16 ± 1.41 ng/dL *vs* post 21.06 ± 1.86 ng/dL; *p* < 0.001). SHBG increased significantly from 26.3 ± 7.9 to 34.1 ± 8.7 nmol/L; *p* < 0.001 [60].

Moreover, according to Cincione and colleagues, KDs determine a reduction in blood glucose levels, insulinemia and an improvement in insulin sensitivity, which consequently led to a reduction in androgen production, whereas the contextual reduction of FM reduced the acyclic production of estrogens deriving from the aromatization in the adipose tissue of the androgen excess, with an improvement of the LH/FSH ratio [69]. This last ratio improves through the reduction of the excess of LH thanks to a relative increase in FSH. Lastly, KDs resulted in an increase in SHBG with a consequent reduction in bioactive free testosterone, thus contributing to a further improvement of hyperandrogenism [69].

### Ketogenic Diet and Postmenopause

Menopause is a physiological condition that is diagnosed in the absence of menses for at least 1 year [74]. Indeed, according to Landgrend and colleagues, clinical menopause is diagnosed when a woman has not menstruated for 12 months due to the loss of ovarian follicular activity [75].

During a woman’s fertile life, the average level of total estrogens is 100–250 pg/mL [76]. On the other hand, the concentration of circulating E2 decreases to 10 pg/mL after menopause. Such drastic hormonal reduction is associated with pathological conditions related to menopause, such as sleep disorders, urogenital atrophy, osteopenia and osteoporosis, sexual dysfunction, CVD, cancer, metabolic disorders and obesity [77]. In particular, women display increased risk of developing CVD after menopause due to estrogen deficiency and dysregulation of lipid metabolism [78].

According to Yasui and colleagues, menopausal symptoms, bone loss, changes in lipid profiles and reduction of insulin sensitivity, are frequently observed in women during the menopausal transition, due to an abrupt decrease in circulating estrogen level [79]. On the other hand, circulating levels of testosterone and dehydroepiandrosterone sulfate gradually decrease with age in postmenopausal women, although transient increases have been observed during the menopausal transition [79]. In fact, transient increases of testosterone level have been suggested to be associated with increased risk of CVD, increased triglyceride, and metabolic syndrome [80].

Moreover, menopause induces an increase in body weight, with redistribution of lipids from subcutaneous to visceral depot, resulting in increased inflammatory markers and circulating levels of low-density lipoprotein cholesterol (LDL-C) and reduced insulin sensitivity [81]. Indeed, as reported by Ko and Kim, estrogens, particularly E2, exerts a protective role on the cardiovascular system and is mainly produced in the ovaries through a process involving LDL-C as a substrate [76]. During menopause, circulating LDL-C cannot be used in estrogens synthesis, resulting in decreased estrogens production. Therefore, menopause is associated with increased LDL-C circulating levels and increased CVD [76]. Metabolic alterations associated with weight gain and fat accumulation in menopause increase the risk of metabolic syndrome, as well as cardiovascular and neurodegenerative diseases, induce an increase in peripheral tissues aromatase activity and promote systemic inflammation, synergizing with age-related processes [82, 83]. The application of ketogenic protocols such as very low energy diet (VLED) and VLCKD to postmenopausal women with overweight or obesity is able to promote the reduction of total body weight and visceral adipose tissue with a concomitant preservation of lean mass [84]. In the TEMPO Diet Randomized Clinical Trial, 101 postmenopausal women, aged 45 to 65 years with BMI from 30 to 40 kg/m^2^, who were at least 5 years after menopause, were recruited. Participants were randomized to either 12 months of moderate (25–35%) energy restriction with a food-based diet (moderate intervention group) or 4 months of severe (65–75%) energy restriction with a total meal replacement diet followed by moderate energy restriction for an additional 8 months (severe intervention group). Compared with the moderate group at 12 months, the severe group lost more weight (effect size, − 6.6 kg; 95% CI, − 8.2 to − 5.1 kg); moreover, whole-body FM (effect size, − 5.5 kg; 95% CI, − 7.1 to − 3.9 kg), abdominal subcutaneous adipose tissue (effect size, − 1890 cm^3^; 95% CI, − 2560 to − 1219 cm^3^), and visceral adipose tissue (effect size, − 1389 cm^3^; 95% CI, − 1748 to − 1030 cm^3^) loss were also greater for the severe group than for the moderate group at 12 months [84].

Ford and colleagues analyzed data from the Women’s Health Initiative Observational Study, considering four dietary patterns among postmenopausal women aged 49–81 years [mean 63.6 (SD 7.4) years]: a low-fat diet, a reduced-carbohydrate diet, a Mediterranean-style diet and a diet consistent with the US Department of Agriculture’s Dietary Guidelines for Americans (DGA) [85]. At the end of the study, the reduced-carbohydrate diet was inversely related to weight gain (OR 0.71; 95% CI 0.66 to 0.76), whereas the low-fat (OR 1.43; 95% CI 1.33 to 1.54) and DGA (OR 1.24; 95% CI 1.15 to 1.33) diets were associated with increased risk of weight gain. By baseline weight status, the reduced-carbohydrate diet was inversely related to weight gain among women who were normal weight (OR 0.72; 95% CI 0.63 to 0.81), overweight (OR 0.67; 95% CI 0.59 to 0.76) or class I obesity (OR 0.63; 95% CI 0.53 to 0.76) at baseline. The low-fat diet was associated with increased risk of weight gain in women who were normal weight (OR 1.28; 95% CI 1.13 to 1.46), overweight (OR 1.60; 95% CI 1.40 to 1.83), class I obesity (OR 1.73; 95% CI 1.43 to 2.09) or class II obesity (OR 1.44; 95% CI 1.08 to 1.92) at baseline. The authors concluded that a low-carb diet can reduce the risk of weight regain in postmenopausal women compared to a low-fat diet [7].

Accordingly, KDs reduced fasting glycemia, fasting insulin, homeostasis model assessment of insulin resistance: in fact, the severe reduction of carbohydrates, leading to a reduction in plasma insulin levels, induces a reduction in lipogenesis processes in favor of lipolytic ones, resulting in a massive utilization of adipocyte triglycerides for energy purposes [86].

In fact, Boden and colleagues conducted a study enrolling patients with T2DM and obesity, demonstrating that the application of KD for 2 weeks induced the reduction of fasting glycaemia from 7.5 to 6.3 mmol/l and the increase of insulin sensitivity by 75% [87].

Additionally, KDs, particularly VLCKD determines a reduction of insulin levels and a concomitant improvement in plasma lipids by inhibition of HMG-CoA reductase [88, 89]: in fact, the improvement of insulin resistance has positive effects on lipid metabolism through the action on 3-hydroxy-3-methylglutaryl coenzyme A reductase and striking effects on lipoprotein size and subclass particle concentrations [30]. Furthermore, KDs promote the reduction of inflammatory pathways [89–91]: according to Paoli and colleagues, that beta-hydroxybutyrate (β-OHB) inhibits NLRP3/inflammasome activation [92]. In fact, β-OHB inhibits NLRP3/inflammasome activation through the reduction of K^+^ efflux from macrophages and the inhibition of the inflammasome assembly. Moreover, β-OHB-dependent inhibition of IL-1β and IL-18 secretion in human monocytes has been documented. Lastly, KDs could promote the reduction of reactive oxygen species (ROS) in vitro and in vivo [93]. According to Stafford and colleagues, KD reduced ROS production in tumor cells. Gene expression profiling demonstrated that the KD induces an overall reversion to expression patterns seen in non-tumor specimens. Notably, genes involved in modulating ROS levels and oxidative stress were altered, including those encoding cyclooxygenase 2, glutathione peroxidases 3 and 7, and periredoxin 4. To demonstrate the ability of ketones to quantitatively reduce ROS in cultured GL261 cells, the authors treated them with either 2 mM β-OHB/acetoacetate or 10 mM β-OHB/acetoacetate for 24 h prior to ROS analysis using 20 μM 2′, 7′-dichlorofluorescein diacetate (DCF). Tumor cells had high levels of ROS as determined by DCF fluorescence, and the application of either 2 mM or 10 mM ketones resulted in a statistically significant (*p* < 0.001) decrease in the DCF signal, demonstrating the quantitative reduction of ROS in these cell [93].

## Ketogenic Diets in Main Disorders of the Female Reproductive Tract

### Ketogenic Diets and PCOS

PCOS is the most common endocrine disease among women in reproductive age, affecting 10 to 15% of women worldwide [94]. The diagnostic criteria include two out of three features: hyperandrogenism, polycystic ovaries on ultrasound and menstrual irregularities [95]. In addition to hormonal changes in gonadotropins and estrogens, obesity, insulin resistance with compensatory hyperinsulinemia and a chronic low-grade inflammatory state often coexist in PCOS [96]. In fact, in women with PCOS, LH concentration increases in relation to FSH, resulting in excessive androgen production. Physiologically, FSH stimulates ovarian follicle maturation and estrogen secretion. In addition, FSH also increases the activity of aromatase, the enzyme responsible for the conversion of androgens into estrogens [96]. Furthermore, insulin acts by increasing the production of androgens (with direct action on the theca cells) and decreasing the hepatic synthesis of the main testosterone-binding protein, resulting in testosterone circulating in the unbound active form. According to Shirazi and colleagues, women with PCOS have abnormal gonadotropin concentration and great androgen biosynthesis from the adrenal and ovaries, stimulated by high levels of insulin, irrespective of body weight [97]. Both metabolic and endocrine alterations of PCOS contribute to the development of metabolic syndrome, T2DM and infertility [98].

Dietary intervention and physical activity also have a positive influence in reducing insulin resistance, body weight and low-grade chronic inflammation. Despite the importance of lifestyle modifications, to date there is still no consensus regarding preferable nutritional treatment for women with PCOS. Since obesity worsens the clinical presentation of PCOS, according to the recommendations of the International Evidence-based Guideline for the Assessment and Management of PCOS, weight management is the main treatment strategy in women with obesity and PCOS. Recent studies have shown that a VLCKD can lead to weight loss and improved insulin sensitivity in PCOS [99, 100]. Paoli and colleagues showed that KD, through improvement of hyperinsulinemia and body composition, contributed to the normalization of the clinical feature in PCOS [60]. Mavropoulos and colleagues conducted a pilot study investigating metabolic and endocrine effects of a low-carbohydrate, KD on overweight and women with obesity and PCOS for 6 months [70]. The authors reported that LCKD showed a significant reduction in body weight (− 12%), free testosterone (− 30%), LH/FSH ratio (− 36%), and basal insulin − 3%) [70].

Hence, improved lifestyle, attention to diet and body weight control are essential building blocks for fertility. According to Barrea and colleagues, high carbohydrate intake and low-grade inflammation influence the development of insulin resistance and hyperandrogenism, thus influencing the pathophysiology of PCOS [1].

According to recent studies, insulin resistance is a condition present in both women with PCOS and with or without obesity [101–103].

Another important effect of KDs for PCOS is the activation of AMPK and SIRT-1, even in the absence of caloric deprivation. Once activated, SIRT1 and AMPK produce beneficial effects on glucose homeostasis and improve insulin sensitivity [60].

**Fig. 1:**
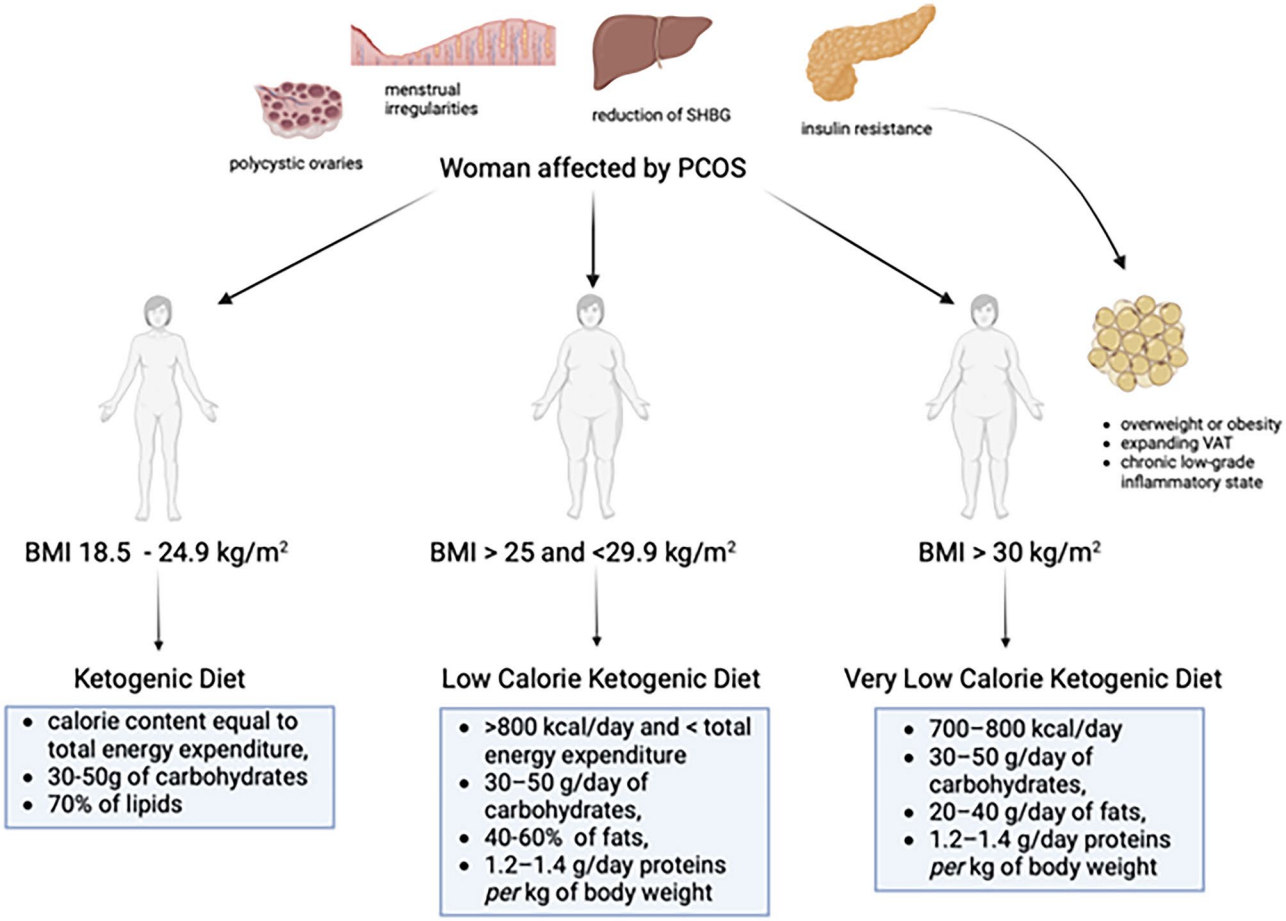
Different types of ketogenic diet applicable in women with PCOS, depending on body weight

When fertility represents the main target of PCOS patients, particularly at more advanced ages, it is critical to rapidly improve metabolic, nutritional and endocrine conditions in order to facilitate ovulation and pregnancy. KDs have proven higher efficacy in terms of rapidity of weight loss, dampening of insulin resistance and induction of ovulation, when compared to standard hypocaloric diets. In order to effectively tailor dietary treatment, it would be appropriate to apply different types of KDs, depending on body weight, BMI, body composition with particular attention to the percentage of adipose tissue, as reported in Fig. 1. In particular, when obesity or overweight are present, VLCKD represents an extremely effective nutritional tool to obtain a rapid and persistent weight loss and hence improve all clinical conditions in patients with PCOS.

### Ketogenic Diets and Tumors

According to recent evidence, cancer represents the second cause of death worldwide, after CVD, in the total population for both sexes combined, and the most commonly diagnosed cancer worldwide is female breast cancer [104, 105]. In 2018, breast cancer represented the first cause of cancer-related death in women worldwide [106]. Interestingly, in 2017, the Center of Disease Control estimated that 40% of all cancers were caused by overweight and obesity (55% in women and 24% in men) [107]. Accordingly, the excess of adipose tissue increases the risk of death in both premenopausal and postmenopausal patients with breast cancer, decreasing overall survival and reducing the effectiveness of cancer treatments [7, 108]. Distinguishing the breast cancer burden by menopausal status is of particular interest, due the opposing role of estrogens in influencing breast cancer development in obesity [109]. In premenopausal women, obesity is associated with a lower risk of estrogens receptor positive (ER^+^) breast cancer and a higher risk of triple‐negative breast cancers (ER, progesterone receptor, PR, and human epidermal growth factor receptor 2 (HER2). On the other hand, in postmenopausal women, obesity is associated with a markedly higher risk of ER^+^ breast cancer [110]. The different influence of obesity on ER^+^ breast cancer development, depending on menopausal status, is explained by hormonal changes, in terms of circulating estrogen, before and after menopause [111, 112]. Ovarian 17β-estradiol (E2) is the main circulating estrogen in the reproductive age. After menopause, E2 circulating levels are markedly reduced and estrone (E1) represents the primary form of estrogen, resulting from the conversion of adrenal androstenedione by aromatase, occurring in peripheral tissues, including adipose tissue and breast. Of note, adipose tissue is the major component of the postmenopausal breast and increased aromatization is observed in obese women [113]. Moreover, it is well known that dysfunctional adipocytes promote low-grade inflammation in obesity and cancer-associated adipocytes recruit and activate macrophages through CCL2/IL-1β/CXCL12 signaling pathway, promoting in turn stromal vascularization and angiogenesis and facilitating tumor progression [114, 115]. It is important to highlight that those patients with breast cancer gain weight even after the diagnosis of cancer, losing muscle mass and developing sarcopenic obesity, displaying negative prognostic effect [116]. Ovarian and endometrial cancer are also associated with obesity, especially in postmenopausal women, due to hormonal changes affecting insulin and IGF-I, all of which are increased in obesity [117]. Although treatment against cancer is mostly based on radio and chemotherapy, there is a great attention to non-pharmacological therapies, including diet therapy, as a tool to modify the cellular metabolism of cancer cells [118]. Growing scientific evidence defines cancer as a metabolic disease [117]. Indeed, the high rate proliferation of cancer cells is mostly related to glucose metabolism than oxygen consumption; the shift from oxidative phosphorylation to anaerobic glycolysis (i.e., the Warburg Effect) is considered as a hallmark of cancer cells [119]. KDs has been applied for over 80 years as an effective adjuvant therapy in refractory epilepsy [120]; however, since 1990s, KDs has been also used to treat metabolic disease (such as obesity and T2DM), CVD cancer [121]. The therapeutic potential of KDs in oncology is related to their ability to impair mitochondrial function, reduce the synthesis of reactive oxygen, decrease chronic inflammation and delay tumor growth, angiogenesis and vascularization of tumor environment [122, 123]. Considering the relevant contribution of inflammation to cancer development, the use of a diet or dietary components (omega 3 fatty acids, ketone bodies, soluble fiber and resistant starch), that reduces inflammation, may reveal as precious tools in cancer prevention and treatment [124]. Finally, KD protocols ensure a valid protein supply in order to satisfy the metabolic needs of the patients, causing weight loss without losing lean body mass. This aspect is critical to maintain performance status in patients with cancer, and to avoid malnutrition, especially in the context of oncological treatments [118].

#### Ketogenic Diets and Breast Cancer

**Fig. 2:**
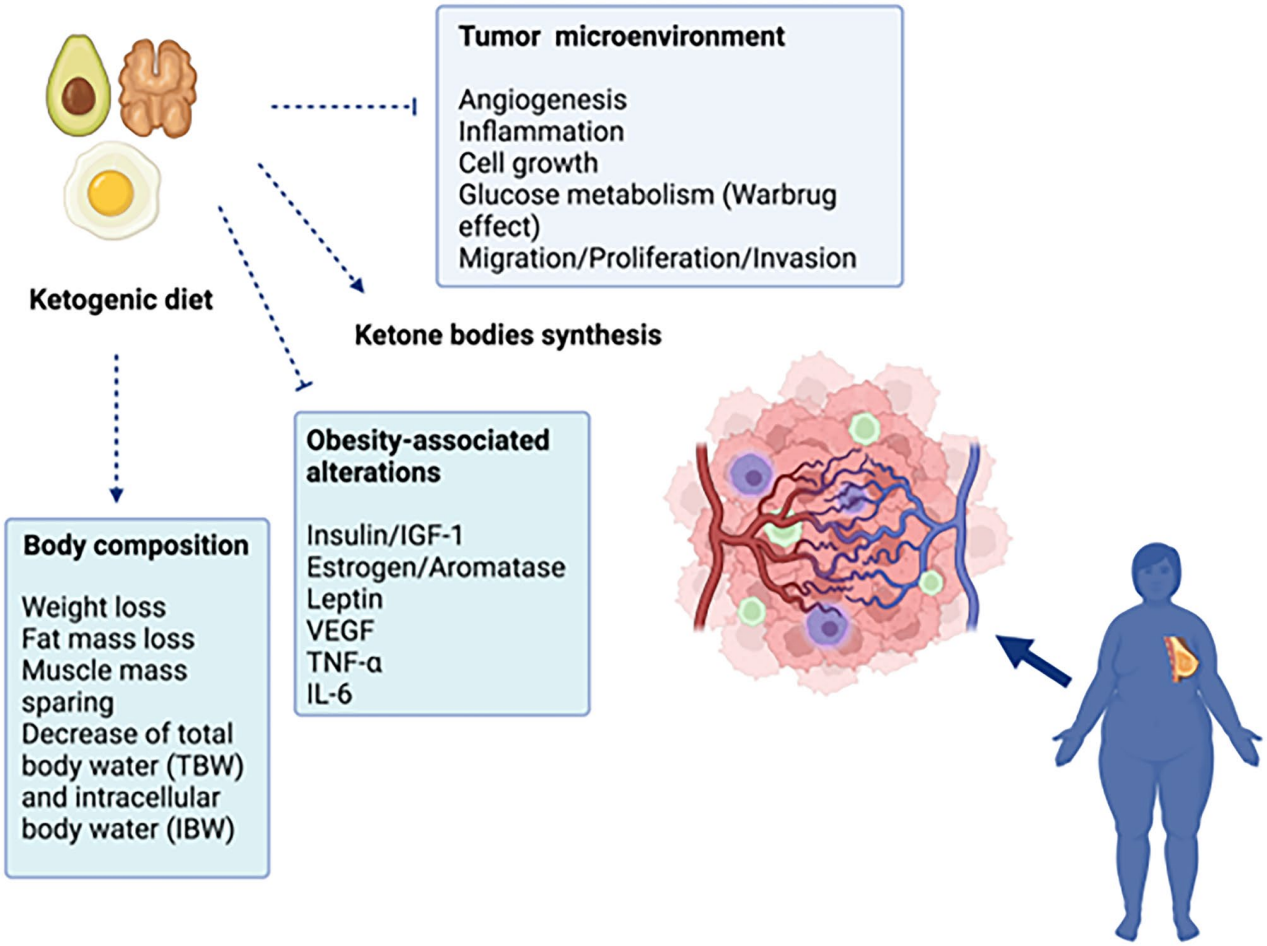
Mechanisms of ketogenic diets on cancer cell in woman with obesity affected by breast cancer. Blue cells represent macrophages, green cells lymphocytes, pink cells cancer cells. Arrows indicate activation. Truncated lines indicate inhibition

Breast cancer represents a significant cause of morbidity and mortality in women, as already mentioned [104]. According to the American Cancer Society, breast cancer alone contributes to 30% of all female cancers [125]. Several trials investigated the role of KDs as an adjuvant therapy to enhance sensitivity to chemotherapy and radiotherapy in locally recurrent or metastatic breast cancer [126•, 127]. Studies confirmed the feasibility and the safety of KDs in patients affected by breast cancer with positive effects on body composition, physical performance, and quality of life during the rehabilitation phase [128, 129]. However, most of these studies do not use standardized KDs therapeutic protocols, and the nutritional patterns are very heterogeneous, both in terms of ketogenic ratio and calorie intake [130]. VLCKD, through its drastic calorie restriction and very low-carbohydrate content, reduces glucose availability in cancer cells, impairing the pro-tumorigenic microenvironment and promoting the death of tumor cells through a pro-apoptotic mechanism [123], as represented in Fig. 2.

However, in vivo, the effectiveness of VLCKD in the treatment of breast cancers has been poorly explored. In this scenario, VLCKD may represent a valuable tool for female cancer prevention in women with obesity; moreover, it could be a valuable tool in women with overweight/obesity and a previous history cancer, in order to reduce body weight, visceral adipose tissue, inflammation and the subsequent risk of recurrence. VLCKD represents a time-limited (~ 12 weeks) nutritional pattern that mimics fasting through a marked restriction of daily carbohydrate intake (lower than 30 g/day), with a daily protein intake approximately 1.2–1.5 g/kg of ideal body weight, a fixed amount of fat (20 g/day, mainly from olive oil and omega-3 fatty acids), and a total daily energy intake < 800 kcal [21].

In patients with previous breast cancer, it would also be interesting to assess the VLCKD protein quality favoring vegetable proteins; in fact, according to Pan and colleagues, higher vegetable protein intake was associated with lower breast cancer incidence and lower risk of death after breast cancer, decreasing cancer-related growth factors, such as insulin and IGF-1 [131].

#### Ketogenic Diets and Endometrial Cancer

Endometrial cancer is the sixth most prevalent cancer in women worldwide [132]. As reported by Braun and colleagues, main risk factors for endometrial cancer are represented by unopposed estrogens therapy, early menarche, late menopause, infertility or failure to ovulate, and PCOS [133]. Additional risk factors are increasing age, obesity, hypertension, T2DM, nulliparity, smoking and hereditary non-polyposis colorectal cancer [133]. According to Onstad and colleagues, the incidence and mortality of endometrial cancer continue to grow, and this trend is a result of the worldwide obesity epidemic [134]. Indeed, more than half of endometrial cancers are currently attributable to obesity, which is recognized as an independent risk factor for this disease [134]. In fact, visceral adipose accumulation promotes proinflammatory milieu, along with deficiency of protective immune cell types in the endometrium, potentially contributing to endometrial cancer risk [135]. Moreover, adipose tissue is responsible for peripheral aromatization of adrenal androgens to estrogens, which, in turn, stimulates endometrium proliferation [8]. During the reproductive stage, such effect is regulated by cyclical progesterone release and regular menstrual bleeding. On the other hand, physiological progesterone decrease, occurring after menopause, together with fat accumulation, contributes to unopposed hyper estrogenic state in obesity, thus increasing the risk of endometrial carcinogenesis [136] (Fig. 2). In addition, evidence of a causal association between obesity-related hyperinsulinemia and endometrial cancer has been provided. Endometrial cancer cells express insulin receptors and both insulin and IGF-1 display mitogenic and antiapoptotic activity, thus potentially contributing to progression of cancer [137, 138]. Accordingly, elevated fasting insulin levels are associated with increased prevalence of endometrioid adenocarcinoma in postmenopausal women. Indeed, elevated insulin circulating levels induce a reduction in SHBG and IGF-binding proteins availability, thus promoting endometrium stimulation by free estrogens and IGF-1 [139].

Given the well-established cancer cells dependence on glucose and insulin availability, KDs is a valid strategy, in association with pharmacological treatment, to counteract cancer proliferation [140]. In fact, glucose and insulin deprivation occurring in patients undergoing a KDs promotes a metabolic environment, which is not suitable for cancer cells growth [141]. In 2018, a randomized controlled trial examining the effects of a 12-week KD in women with ovarian or endometrial cancer, demonstrated for the first time that KD is effective in promoting visceral fat loss, maintaining lean body mass, and decreasing cancer-related growth factors, such as insulin and IGF-1 [117]. Importantly, the same group found that KD eases symptoms due to cancer-related fatigue, along with improved physical function, increased energy, and reduced specific food cravings, thus improving the quality of life in women with ovarian or endometrial cancer [142].

Furthermore, according to Kokts-Porietis and colleagues, obesity at the time of endometrial cancer diagnosis was associated with increased recurrence and all-cause mortality among endometrial cancer survivors [143]. In this context, VLCKD may represent a preventive measure in postmenopausal women with obesity, to reduce visceral adipose tissue, inflammatory status, growth factors such as insulin and IGF-1, and to reduce the risk of endometrial cancer. Moreover, it could be a valuable tool in women with prior endometrial cancer and obesity to reduce body weight, visceral adipose tissue, circulating insulin levels and risk of recurrence.

## When is it Possible to Set Up a VLCKD Protocol?

According to Consensus Statement of Italian Society of Endocrinology (SIE), short- and medium-term studies support the use of VLCKD in individuals with obesity (BMI ≥ 30.0 kg/m^2^) or overweight (BMI 25.0–29.9 kg/m^2^) with abdominal obesity (WC > 88 cm in women) [21]. In addition, VLCKD may be recommended when excess body weight is related to comorbidities including T2DM, NAFLD, obstructive sleep apnea syndrome, and PCOS [20, 21].

Conversely, some conditions represent absolute contraindications to the use of VLCKD. In particular, pregnancy and lactation, or childhood and adolescence are physiological conditions during which VLCKD should be avoided. Pathophysiological conditions include the presence of comorbidities such as hepatic, renal, cardiac and respiratory insufficiency, type 1 diabetes, myocardial infarction or recent cerebrovascular stroke, severe psychiatric disorders. In addition, eating disorders and alcohol and substance abuse are contraindications for VLCKD [20, 21].

## VLCKD Protocol: Step by Step

As stated in the SIE position statement, the VLCKD protocol is divided into different stages, involving the drastic reduction of carbohydrate content for the first 8–12 weeks, to induce nutritional ketosis [21]. The initial stage of VLCKD is characterized by a very low-calorie diet (650–800 kcal/day), low in carbohydrate (< 30 g daily from vegetables), and fat (only 20 g per day, derived also from olive oil). The amount of high-biological-value protein ranged between 1.2 and 1.4 g per each kg of ideal body weight in order to preserve lean mass: protein can be derived from conventional foods (such as eggs, meat or fish) or meal replacements can be used [21, 23]. Scientific evidence suggests that meal replacement in the first active ketogenic phase should be recommended to ensure a safe, effective, and controlled administration of VLCKD [30]. In fact, with the use of single-portioned meal replacement meals, the calibration of the diet is more accurate, and the content of calories, macronutrients and micronutrients needed by the patient can be set more precisely and individually [30].

Therefore, it would seem more appropriate to set up a VLCKD protocol with the use of meal replacements to determine greater safety, efficacy, and compliance in patients with obesity, with preference given to freeze-dried meal replacements, which generally have a higher protein content and lower fat and carbohydrate content. This would ensure a higher degree of weight loss and a better adherence [22].

Being a very low caloric nutritional pattern, it is recommended to supplement patients with micronutrients (vitamins, such as complex B vitamins, vitamin C and E, minerals, including potassium, sodium, magnesium, calcium; and omega-3 fatty acids) according to international recommendations [22]. Hydration is very important at this early stage: about 2–2.5 L of water per day are needed. In addition, it is very important to use low glycemic index vegetables to reach the necessary fiber quota [22]. After this first active ketogenic phase, low-calorie diet is prescribed (LCD stage): at this point, different food groups will be progressively reintroduced. In particular, carbohydrates are gradually reintroduced, starting from foods with the lowest glycemic index (such as dairy products and fruits). The daily calorie intake of LCD diet ranges between 1000 and 1200 kcal/day, with carbohydrates amount of 60–100 g. Then, a hypocaloric balanced diet is sustained, with a caloric intake between 1300 and 1400 kcal and a carbohydrate intake of 130–150 g; legumes are reintroduced [22].

Lastly, a maintained hypocaloric balanced diet is sustained, following a Mediterranean diet with a caloric intake between 1500 and 1800 kcal, with the reintroduction of low glycemic index cereal. This last step, through the acquisition of correct eating habits, is crucial for maintaining long-term results [22]. As reported, it is essential for the patient with obesity to lose at least 15% of body weight and maintain this loss over the long term to reduce cardiometabolic risks [22, 23]. Energy intake is provided by 30% fat, 45% carbohydrate, and 25% protein, as for the traditional Mediterranean diet. More in detail, protein intake should be 1–1.2 g/kg of desirable weight (desirable weight: weight corresponding to a BMI of 22.5 kg/m^2^). Recommended carbohydrate sources should be high-fiber foods with slow-absorption starches, avoiding high simple sugars-intake (max 10%) and favoring the intake of vegetables, fruit, cereals, and legumes. This nutritional profile allows patients to continue their nutritional re-education while maintaining the achieved weight loss [22, 23].

Finally, we emphasize that both the KD and the Mediterranean diet have specific advantages for endocrine disorders of the female reproductive system. As we have previously seen, KD can promote weight loss, improve insulin sensitivity, and potentially improve hormonal balance, but it can cause nutrient deficiencies, limit food choices, and lack long-term research [144]. The Mediterranean diet offers a nutrient-rich approach, is beneficial for heart health and has anti-inflammatory properties, but may have limited effects on weight loss, requires individual variation and has risks related to processed foods [145]. However, the two nutritional approaches can be combined to enhance beneficial effects for patients with endocrine disorders of the reproductive system. Very recently, Verde and colleagues demonstrated in 318 women with overweight/obesity that a high adherence to the Mediterranean diet prior to the start of VLCKD improves the efficacy of the latter in terms of both weight loss and improvements in body composition [146•]. The authors attributed these results to the presence of bioactive compounds in the Mediterranean diet, which together and in the context of Mediterranean diet could lead to a favorable metabolic set-up for the onset of more effective ketosis [146•]. Furthermore, it is important to remind that a Mediterranean-style nutritional approach represents the last step (maintenance phase) of the VLCKD protocol [23]. For this reason, both nutritional approaches could be appropriately combined in women with endocrine disorders of the reproductive system to maximize the beneficial effects of the dietary intervention as part of their management.

## Conclusions

As inflammatory status, oxidative stress and excess adipose tissue could have a negative effect on fertility and are associated with the development of female diseases including PCOS and cancer, finding successful therapeutic strategies is of utmost importance. Furthermore, given the preeminent role of obesity as significant risk factor for the development of endocrine disease in women, any therapeutical strategy should include specific dietary guidelines aimed at reducing inflammatory parameters. The use of KDs has shown significantly favorable effects in reducing inflammation and achieving weight loss, particularly FM and visceral adipose tissue. Ketogenic protocol is effective in nutritional and cardiometabolic rehabilitation in various pathophysiological conditions in women, particularly menopause, cancer and insulin resistance. KDs should be prescribed under close medical supervision. Furthermore, the diet should be tailored to the individual patient, customizing the calorie and macronutrient content. Since there are few published studies on the effect of KDs on PCOS, fertility, and breast and endometrial cancers (Table 1), well-designed controlled studies are needed to establish the best KD protocol in terms of diet period, macro- and micronutrient combinations, use of supplements, and carbohydrate reintroduction.

**Table 1:** Studies on the effects of KDs in main endocrine diseases of the female reproductive system

**Ref**	**Study design**	**Aim**	**Participants**	**Age**	**BMI**	**Type and duration of KD**	**Main findings**
***PCOS***							
*Paoli et al.* [60]	Single-arm clinical trial	To determine the effects of KD in women of childbearing age with a diagnosis of PCOS	24 women with overweight and with PCOS	18–45 years	≥ 27 kg/m^2^	Mediterranean eucaloric ketogenic protocol: 1600/1700 kcal/day, fat 71.1 ± 9.3% TE, protein 24.1 ± 5.6% TE, CHO 24.1 ± 5.6% TE 12 weeks	Significant reductions of anthropometric and body composition parameters. Significant decrease in glucose, insulin, and consequent significant improvement of HoMA-IR. Significant decrease of TG, total cholesterol and LDL along with a rise in HDL cholesterol levels. The LH/FSH ratio, LH, total testosterone, free testosterone and DHEAS were also significantly reduced. FSH values were found modestly increased. Estradiol, progesterone and SHBG significantly increased
*Mavropoulos et al. *[70]	Single-arm clinical trial	To investigate metabolic and endocrine effects of KD on subjects with overweight or obesity and with PCOS	5 women with overweight and with PCOS	18–45 years	≥ 27 kg/m^2^	Low-carbohydrate, ketogenic diet: less of 20 gr/die of CHO and no restriction in protein and fat intake 6 months	Significant reductions from baseline to 24 weeks in body weight, % free testosterone, LH/FSH ratio, and insulin. 2 women became pregnant despite previous infertility problems
**Breast cancer**							
*Khodabakhshi et al. *[126•]	RCT	To evaluate the effects of a KD in patients with locally advanced and metastatic breast cancer receiving chemotherapy	60 patients with breast cancer undergoing treatment with chemotherapy (*n* = 30 intervention group, *n* = 30 control group)	18–70 years	Not reported	Eucaloric MCT based KD: CHO 6% TE, protein 19% TE, MCT 20% TE and fat 55% TE 90 days	TNF-α decreased significantly, while IL-10 increased in the intervention compared to the control group. Patients in the KD group had lower adjusted serum insulin compared to the control group. KD lead to a higher reduction in tumor size compared to the control diet. Stage decreased significantly in patients with locally advanced disease in the KD group compared to control group
*Kämmerer et al. *[128]	Open-label non-randomized trial	To compare 3 diet types (healthy standard diet, KD and low CHO diet) and to assess feasibility, safety, and tolerability in patients with breast cancer	121 patients with breast cancer (*n* = 25 healthy standard diet group, *n* = 20 KD group, *n* = 76 low CHO diet group)	26–69 years	Mean BMI healthy standard diet group 26.6 kg/m^2^, KD group 23.4 kg/m^2^, low CHO diet group 27.2 kg/m^2^	Modified Atkins diet ad libitum: CHO 2–4% TE, protein 16–18% TE and fat 80–85% TE 20 weeks	Regard to physical performance, the respiratory quotient in the KD group decreased to 0.75 (0.65–0.83), almost reaching the 0.7 value of pure fat oxidation, and was therefore significantly lower than the respiratory quotient in the low CHO diet and healthy standard diet groups. Despite being the group with the highest % of advanced diseases, patients in the KD group performed best in the ergometer test at T20, with higher maximum oxygen uptake and maximum workload as well as longer time to exhaustion. Despite increased cholesterol levels, KD patients had the best TG/HDL ratio and HoMA-IR
*Klement et al. *[129]	Non-randomized, controlled trial	To study the effects of KD on quality of life and blood parameters in women with early-stage breast cancer undergoing radiotherapy	59 patients with early-stage breast cancer (*n* = 29 KD group, *n* = 30 standard diet group)	25–70 years	Mean BMI KD group 28.3 kg/m^2^, standard diet group 25.0 kg/m^2^	KD ad libitum: fat 75–80% TE and CHO < 50 g/die 12 weeks	Compared to the standard diet group, women consuming a KD experienced significant improvement in emotional functioning, social functioning, sleep quality, future perspectives, and systemic therapy side effects. While breast symptoms increased significantly in both groups, the increase was less pronounced in the KD group. There was no hint of a detrimental effect of the KDs on either liver or kidney function; in contrast, biomarkers of metabolic health (gamma-glutamyl-transpeptidase, creatinine, TG, IGF-1, FT3) significantly improved in the KD, but not the SD group
**Ovarian and Endometrial cancer**							
*Cohen et al.* [142]	RCT	To assess effects of KD on body composition and lower serum insulin and IGF-1 in women with ovarian or endometrial cancer	45 women with ovarian or endometrial cancer (*n* = 25 KD group, *n* = 20 ACS diet group)	≥ 19 years	≥ 18.5 kg/m^2^	70:25:5 energy from fat, protein, and CHO respectively (12 weeks)	KD group had lower adjusted total and android fat mass compared to ACS group. % of change in visceral fat was greater in the KD group compared to ACS group. KD group had lower adjusted fasting serum insulin compared to ACS group. In KD group there was a significant inverse association between the changes in serum β-hydroxybutyrate and IGF-1 concentrations

*BMI* body mass index, *KD* ketogenic diet, *PCOS* polycystic ovary syndrome, *TE* total energy, *HoMA-IR* Homeostatic Model Assessment for Insulin Resistance, *TG* triglycerides, *HDL* High Density Lipoprotein, *LH* luteinizing hormone, *FSH* follicle-stimulating hormone, *DHEAS* dehydroepiandrosterone sulphate, *MCT* medium chain triglycerides, *TNF- α* tumor necrosis factor, *IL-10* interleukin-10, *IGF-1* insulin like growth factor-1, *FT3* free triiodothyronine, *RCT* randomized controlled trial, *ACS* American Cancer Society
